# Determinants of Obesity and Associated Population Attributability, South Africa: Empirical Evidence from a National Panel Survey, 2008-2012

**DOI:** 10.1371/journal.pone.0130218

**Published:** 2015-06-10

**Authors:** Benn Sartorius, Lennert J. Veerman, Mercy Manyema, Lumbwe Chola, Karen Hofman

**Affiliations:** 1 Discipline of Public Health Medicine, School of Nursing and Public Health, University of KwaZulu-Natal, Durban, South Africa; 2 School of Population Health, University of Queensland, Brisbane, QLD, Australia; 3 School of Public Health, Faculty of Health Sciences, University of the Witwatersrand, Johannesburg, South Africa; 4 PRICELESS SA, MRC/Wits Rural Public, Health and Health Transitions Research Unit, School of Public Health, Faculty of Health Sciences, University of the Witwatersrand, Johannesburg, South Africa; 5 John Hopkins Bloomberg School of Public Health, Johns Hopkins University, Baltimore, United States of America; Leibniz Institute for Prevention Research and Epidemiology (BIPS), GERMANY

## Abstract

**Background:**

Obesity is a major risk factor for emerging non-communicable diseases (NCDS) in middle income countries including South Africa (SA). Understanding the multiple and complex determinants of obesity and their true population attributable impact is critical for informing and developing effective prevention efforts using scientific based evidence. This study identified contextualised high impact factors associated with obesity in South Africa.

**Methods:**

Analysis of three national cross sectional (repeated panel) surveys, using a multilevel logistic regression and population attributable fraction estimation allowed for identification of contextualised high impact factors associated with obesity (BMI>30 kg/m^2^) among adults (15years+).

**Results:**

Obesity prevalence increased significantly from 23.5% in 2008 to 27.2% in 2012, with a significantly (p-value<0.001) higher prevalence among females (37.9% in 2012) compared to males (13.3% in 2012). Living in formal urban areas, white ethnicity, being married, not exercising and/or in higher socio-economic category were significantly associated with male obesity. Females living in formal or informal urban areas, higher crime areas, African/White ethnicity, married, not exercising, in a higher socio-economic category and/or living in households with proportionate higher spending on food (and unhealthy food options) were significantly more likely to be obese. The identified determinants appeared to account for 75% and 43% of male and female obesity respectively. White males had the highest relative gain in obesity from 2008 to 2012.

**Conclusions:**

The rising prevalence of obesity in South Africa is significant and over the past 5 years the rising prevalence of Type-2 diabetes has mirrored this pattern, especially among females. Targeting young adolescent girls should be a priority. Addressing determinants of obesity will involve a multifaceted strategy and requires at individual and population levels. With rising costs in the private and public sector to combat obesity related NCDS, this analysis can inform culturally sensitive mass communications and wellness campaigns. Knowledge of social determinants is critical to develop “best buys”.

## Introduction

Obesity is a global public health concern and the World Health Organisation (WHO) has estimated that it affects 500 million people worldwide with this burden projected to increase to one billion obese globally by 2030 [1,2]. Obesity is associated with significant health risks and comorbidities such as cardiovascular disease, diabetes (type 2) and various cancers [1,3].

Until recently, Sub-Saharan Africa (SSA) was minimally affected by the obesity epidemic due to under-nutrition and a major burden of HIV and tuberculosis [4]. However, in recent years, the African continent has seen a rapid rise in overweight and obesity prevalence as well as associated co-morbidities [5–7]. Within SSA, the prevalence of obesity varies greatly from country to country[8,9]. South Africa is undergoing a rapid epidemiological transition and has the highest prevalence of obesity [4]. Socio-cultural, environmental and behavioural factors including socio-economic status are likely to explain the high prevalence of obesity in South Africa [4,10].

Understanding the determinants of obesity (and their relative population attributability) is crucial for informing policy and developing effective prevention programmes. Such efforts must be based on a detailed scientific understanding of the multiple interlinked risk factors for obesity. The etiology of obesity is multifaceted with factors from multiple contexts, and “discrete” contributions as well as interactions between factors are not yet well understood [10,11]. Attributable fraction methods are helpful tools for public health planning [12] as measures of effect (e.g. odds ratios and relative risks) do not account for prevalence of exposure to a given risk factor and hence “impact” at a population level if a given factor is removed. Individual level survey data also allows the development of discrete exposure matrices to more accurately quantify the true population attributability for a given determinant after the removal of confounding or co-exposure influences of other determinants. This type of data also allows one to test for possible interaction between factors.

This study aims to utilise data from three cross sectional (repeated panel) surveys to assess both prevalence of obesity in South Africa and changes during 2008–2012, identify significant determinants using a multilevel framework, and estimate the discrete population attributable fractions for identified significant determinants. This will inform decisions and will enable policy makers to address obesity in South Africa. A comprehensive multipronged approach is needed to potentially halt and in future reverse the global obesity [11,13].

## Methods

### Data

Data were taken from the three panel (cross-sectional) waves of the South African National Income Dynamics Study (SA-NIDS)[14], the first national panel study in South Africa. SA-NIDS was undertaken by the South African Labour and Development Research Unit based at the School of Economics at the University of Cape Town. The surveys took place in 2008, 2010/11 and 2012. A stratified, two-stage random cluster sample design was employed to sample households for inclusion at baseline and stratified (proportionally allocated) based on the 53 district councils (DCs) in South Africa. Within each DC (primary sampling unit [PSU]), clusters of dwelling units were systematically drawn. A detailed report on the methodology employed in this study is provided elsewhere [15]. The household level response rate was 69% and the individual response rate within households was 93%. The baseline SA-NIDS survey provides data for 28,247 individuals (including children) from 7,301 households. Extensive training of fieldworkers and utilisation of standardised questionnaires across the waves were employed in panel survey to improve/standardise measurements across the different panels. NIDS employed quality controllers to check data completeness/data verification. Both in-field call-backs were utilised to verify interviewer professionalism, ensure that the correct household has been interviewed, obtain key missing data and follow-up on refusals to participate. Telephonic call backs were undertaken by full-time survey assistants. Two random telephonic call backs were done per primary sampling unit (PSU) [15].

### Study population for secondary data analysis

The analysis in this study was restricted to adults aged ≥15 years. The determinants of obesity and population attributable fraction analyses were limited to adult household respondents yielding a final sample size of 7273 respondents in wave 1, 8600 respondents in wave 2 and 9751 respondents in wave 3. Adult household respondents were selected as additional determinant data (e.g. behavioural) were captured for these individuals and not for other individuals within the household.

### Outcome

Weight and height measurements were taken for all individuals as well as waist and blood pressure measurements for all adults in the household. The outcome of interest in this study was obesity (classified as a body mass index [BMI] ≥30 kg/m^2^).

### Determinants

The determinants used in this study were based on existing literature (see theoretical framework below) and were dependent on the variable(s) having been captured in the primary study questionnaires (secondary data analysis limitation). The determinants assessed in this study included: age, gender, ethnicity, education status, marital status, employment status, socio-economic status, behavioural factors (exercise, smoking, alcohol consumption, household expenditure patterns with regards to food and beverages), psychosocial factors (such as depression) and community characteristics (urban [formal or informal] versus non-urban residence, crime). Urban areas are defined as localities (i.e. local municipalities) characterised by a threshold population of greater than 1 000 persons and a population density of greater than or equal to 500 people per square kilometre. Eight background papers were commissioned to inform the design of the NIDS questionnaires. These papers are available on the NIDS website (www.nids.uct.ac.za). The questionnaires were pilot tested on sample households and also involved relevant subject area experts. Depressive symptoms were screened using the 10-item Center for Epidemiologic Studies Depression Scale (CES-D) instrument [16]. A total score of ≥10 for this instrument suggests a positive screen for depressive symptoms. Physical exercise was dichotomous and coded 1 if an individual exercised at least once a week and or 0 if not. There were five smoking-related questions in the adult questionnaire. We used current smoking status in our analyses. The “alcohol” variable was dichotomous and based on whether the respondent drinks less than one day a week (0) or drinks one or more days per week (1). Supine blood pressure and heart rate were measured twice by trained field workers in the left arm after a 5 minute rest period, using an automated blood pressure monitor (Omron M7 BP, multi-size cuff, factory calibrated). An informal settlement is defined as: “unplanned settlements that involve people claiming land and constructing their own housing without legal tenure” [17].

**Fig 1:**
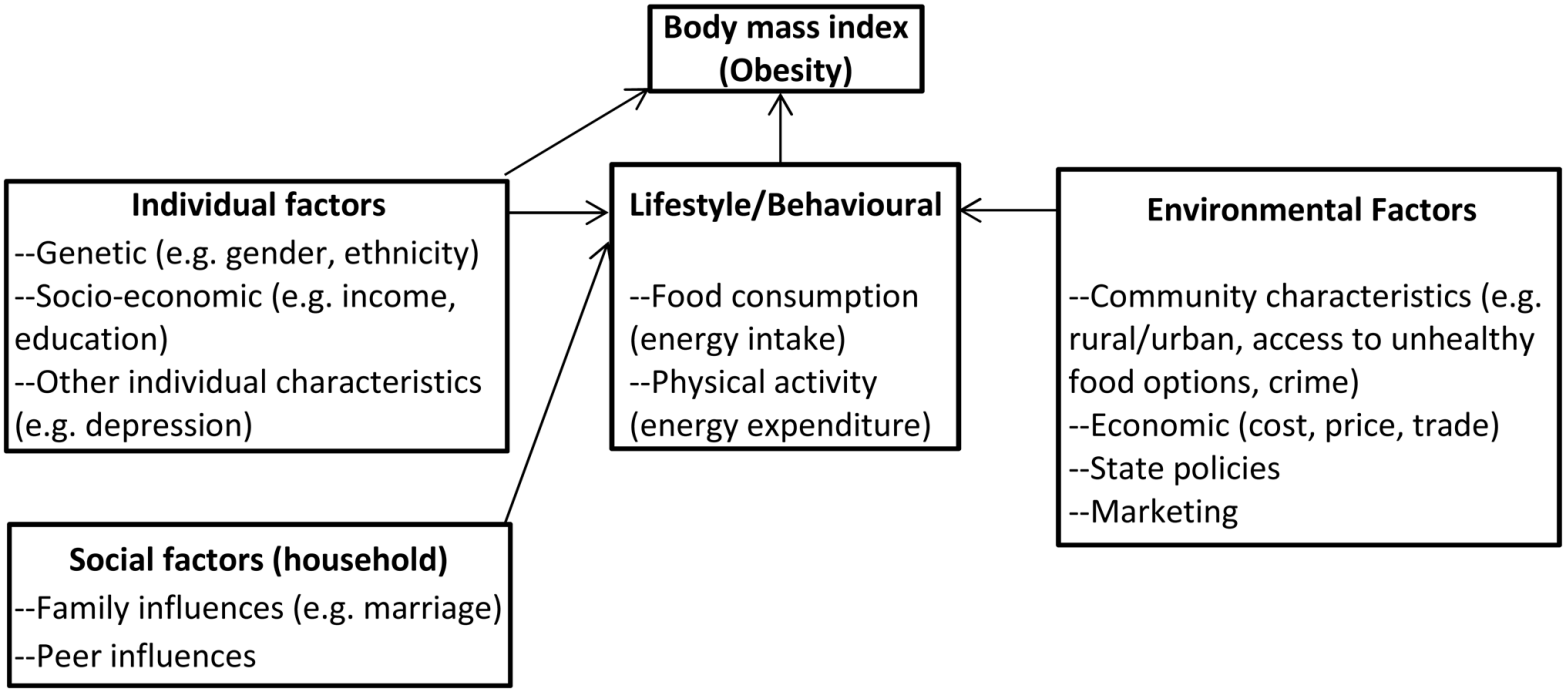
Adapted theoretical framework for available multilevel factors driving adult obesity in South Africa.

The theoretical framework used in this study was adapted from the ecological systems theory[18] and the “sustainable prevention of obesity through integrated strategies” (SPOTLIGHT) project conceptual framework for studying the factors affecting obesity [19–21]–(Fig 1). The ecological theory highlights the importance of environmental factors in the development of individuals. A person is considered to be at the centre of nested structures of the ecological environment: institutions and groups, such as families and neighbourhoods; relations and social ties; cultural contexts; and events and transitions over a life course. Changes in these different levels in the system affect the development of individuals. This multilevel approach to understanding individual development has been used in several studies assessing the influence of various factors on individual health outcomes [22,23]. In the adapted framework below, obesity is a problem arising from a complex system of individual, social-cultural and environmental factors that influence individual behaviours on food consumption and choice (energy intake) and physical activity (exercise and sedentary patterns) i.e. expenditure.

### Statistical analyses

Analyses were performed using Stata software version 12 SE [24]. Clustering as well as survey design effects were accounted for using sample weights to correctly estimate determinant standard error and hence significance.

The mean BMI and proportion of adults who were obese (stratified by gender) were calculated along with 95% confidence intervals [CI’s]. The primary outcome assessed in this study was classification as being obese (BMI≥30 kg/m^2^). Bivariate associations between determinants and the primary outcome were assessed using Chi (χ^2^)-squared statistics (or Fishers exact test in the presence of small cell count i.e. <5). Variables significant at the 20% level (i.e. p-value<0.2) were selected for inclusion in to a multivariable multilevel (individual, household and district) logistic regression using a generalised linear latent and mixed model. Multicollinearity was assessed for the final multivariable models using variance inflation factors (VIF), given that many of the determinants are likely to be highly correlated to one another. Model fit and diagnostics were also run to ensure no violation of assumptions occurred.

### Population attributable risk (AF_p_) calculation

Population attributable fractions (AF_*p*_) are commonly calculated in terms of the prevalence of exposure to a given risk factor in the population and the relative risk (RR) of the outcome for those exposed to that risk factor [25]. We also run the model described above as a Poisson formulation to directly calculate RRs for each of the identified significant determinants for use in the population attributable fraction estimation. This was done because in the presence of a more prevalent outcome (i.e. obesity) the odds ratio from the logistic formulation is likely to substantially overestimate the RR [26]. Use of a Poisson formulation for binary outcome data may however result in overly conservative standard errors (i.e. overestimate significance) due to under-dispersion from fitting a Poisson model to binomial data [26,27]. However, a Poisson approach can be used with robust standard errors to deal with variance overestimation and estimate more correct standard errors [28]. We have used a formula which produces more internal valid estimates when confounding exists [25,29,30]:

$$AF_{p}=pd(\frac{RR-1}{RR})$$

where *pd* refers to the proportion of cases (i.e. obese) exposed to a given risk factor and RR must be an adjusted relative risk [31].

We also estimated the AF*p* for each determinant. The 95% confidence limits for the coefficient are also useful for quantifying the range of AF*p* associated with a given determinant and were estimated based on both the standard errors (95%CI’s) of the coefficients as well as the proportion exposed to that determinant.

## Results

### Descriptive findings

Demographic characteristics of the study population (all adults and respondents) by panel wave are summarised in Table 1. The sample size of adults increased from 18526 in wave (or panel) 1 in 2008 to 22218 individuals in wave 3 in 2012. The majority of adults were female (56%). Most adults lived either in an urban area (42%) followed by tribal authority areas (~38%). The majority of adults were African (~77%) and the majority reported having received some secondary education (~60%). A large proportion of the adult study sample reported never having been married (~46%) followed by married (~24%). The percentage of adult study participants with a valid BMI measurement ranged from 65–74% across the panels.

**Table 1:** Characteristics of the study sample: South-African adults (≥15 years of age) participating in the NIDS Wave 1–3 surveys, 2008–2012.

	Wave (panel) 1		Wave (panel) 2		Wave (panel) 3	
Background characteristic	n	%	n	%	n	%
**Total (adults 15+)**	18526	100.0%	21270	100.0%	22218	100.0%
**Total (adults 15+ with valid ^i^ BMI measurement)**	13771	74.3%	13810	64.9%	15715	70.7%
**Total (adult survey respondents 15+ with valid** ^i^ **BMI measurement)**	7273	39.3%	8600	40.4%	9751	43.9%
**Age**						
15–24	5678	30.6%	5721	26.9%	4930	22.2%
25–34	3659	19.8%	4857	22.8%	5549	25.0%
35–44	3080	16.6%	3571	16.8%	3951	17.8%
45–54	2559	13.8%	3016	14.2%	3260	14.7%
55–64	1773	9.6%	2100	9.9%	2342	10.5%
65+	1777	9.6%	2005	9.4%	2186	9.8%
**Gender**	18300	98.8%	21006	98.8%	21924	98.7%
Female	10351	55.9%	11917	56.0%	12538	56.4%
Male	7949	42.9%	9089	42.7%	9386	42.2%
**Residence**	18526	100.0%	19932	93.7%	21078	94.9%
Rural formal	1922	10.4%	2127	10.0%	2186	9.8%
Tribal authority areas	7247	39.1%	7933	37.3%	8270	37.2%
Urban formal	8096	43.7%	8541	40.2%	9133	41.1%
Urban informal	1261	6.8%	1331	6.3%	1489	6.7%
**Population group**	18526	100.0%	21270	100.0%	22218	100.0%
African	14188	76.6%	16660	78.3%	17409	78.4%
Coloured	2845	15.4%	3080	14.5%	3259	14.7%
Asian/Indian	319	1.7%	340	1.6%	340	1.5%
White	1174	6.3%	1190	5.6%	1210	5.4%
**Education**	18435	99.5%	19223	90.4%	20539	92.4%
No education	2449	13.2%	2366	11.1%	2359	10.6%
Primary	4420	23.9%	4202	19.8%	4265	19.2%
Secondary	11174	60.3%	12292	57.8%	13441	60.5%
Tertiary	392	2.1%	363	1.7%	474	2.1%
**Marital status**	17201	92.8%	17251	81.1%	18780	84.5%
Married	4883	26.4%	4566	21.5%	5139	23.1%
Living with partner	1464	7.9%	1275	6.0%	1419	6.4%
Widow/Widower	1498	8.1%	1450	6.8%	1603	7.2%
Divorced or separated	436	2.4%	385	1.8%	428	1.9%
Never married	8920	48.1%	9575	45.0%	10191	45.9%

^i^: excludes missing values and erroneous extreme values (negative or zero weight or height measurements, extreme BMI values)

**Fig 2:**
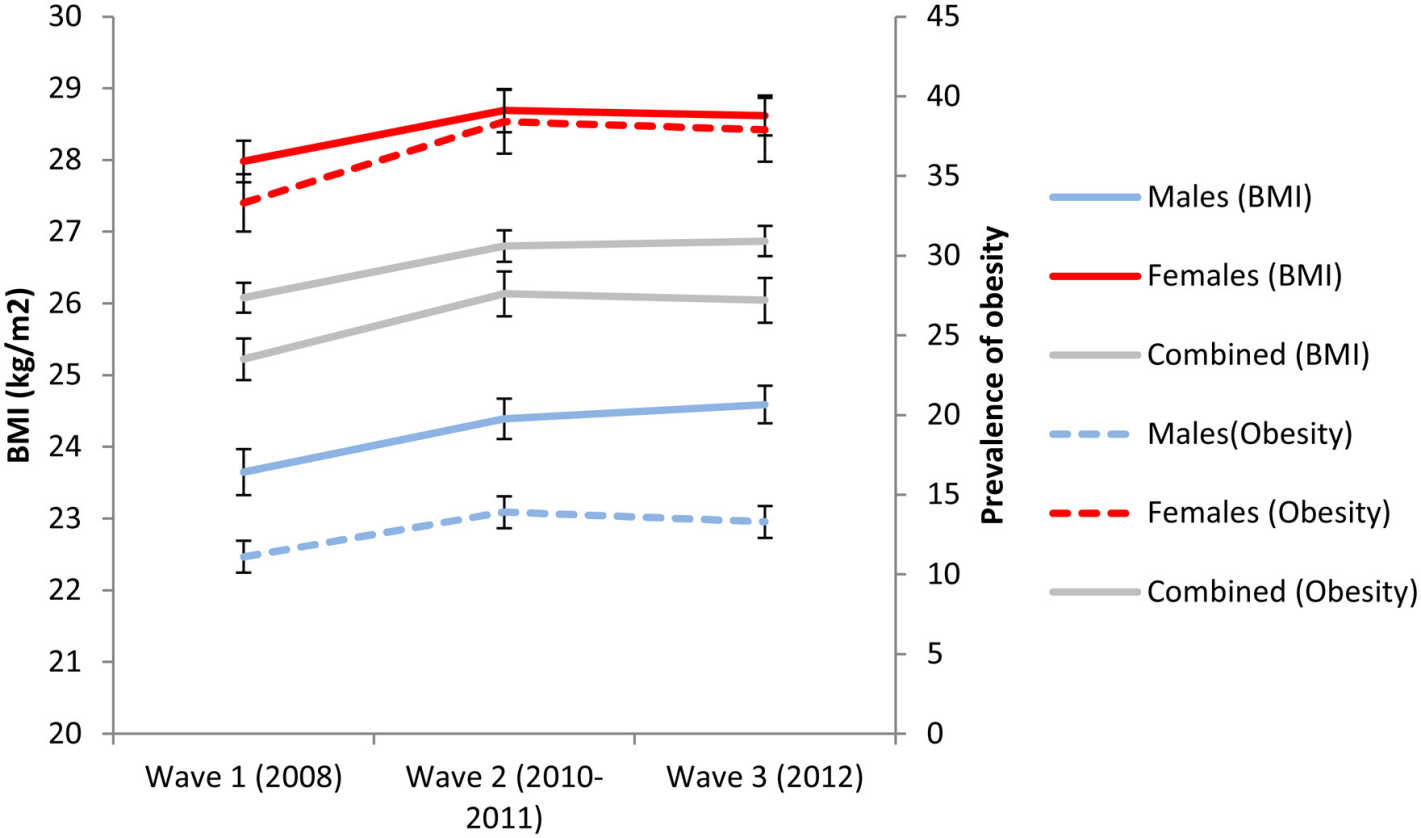
trends in BMI and obesity prevalence by gender and period.

The average BMI and prevalence of obesity increased significantly from 26.1kg/m^2^ and 23.5% in 2008 to 26.9kg/m^2^ and 27.2% in 2010/2011 (Table 2, Fig 2). The data suggests a reduction in the increasing rate of obesity and overweight between waves 2 and 3 (when compared to the significant increase between waves 1 and 2). Females had a significantly higher BMI and prevalence of obesity compared to males in all three panels (p-value<0.001). Female prevalence of obesity ranged from 33.3% in wave 1 to 38.4% in wave 2 compared to 11.1% and 13.9% amongst males. The prevalence of self-reported diagnosed diabetes amongst adults also significantly increased from 3.5% (95% CI: 3.0–4.1%) in 2008 to 5.0% (95% CI: 4.3–5.7%) in 2012.

**Table 2:** Survey weighted anthropometric measurements of all household adults (≥15 years), NIDS Wave 1–3 surveys, 2008–2012.

Measurement	Panel ^i^	Men	Women	Overall
Subjects with valid height and weight measurement	Wave 1	5495	8276	13771
	Wave 2	5542	8267	13809
	Wave 3	6165	9559	15724
Survey weighted mean BMI kg/m^2^ (95% CI)	Wave 1	23.65 (23.33, 23.97)	27.98 (27.69, 28.27)	26.08 (25.87, 26.28)
	Wave 2	24.39 (24.11, 24.67)	28.69 (28.39, 29.00)	26.80 (26.58, 27.02)
	Wave 3	24.59 (24.33, 24.85)	28.62 (28.34, 28.89)	26.87 (26.66, 27.08)
Survey weighted proportions (95% CI):				
Overweight (BMI 25.0 to 29.9 kg/m^2^)	Wave 1	0.203 (0.180, 0.226)	0.263 (0.248, 0.277)	0.236 (0.222, 0.251)
	Wave 2	0.231 (0.208, 0.254)	0.268 (0.254, 0.282)	0.252 (0.238, 0.265)
	Wave 3	0.268 (0.246, 0.291)	0.297 (0.279, 0.315)	0.285 (0.269, 0.300)
Obese (BMI ≥ 30 kg/m^2^)	Wave 1	0.111 (0.093, 0.129)	0.333 (0.315, 0.351)	0.235 (0.222, 0.249)
	Wave 2	0.139 (0.122, 0.156)	0.384 (0.364, 0.405)	0.276 (0.262, 0.291)
	Wave 3	0.133 (0.116, 0.150)	0.379 (0.359, 0.398)	0.272 (0.258, 0.286)

^i^: wave 1 = 2008, wave 2 = 2010/2011, wave 3 = 2012

**Fig 3:**
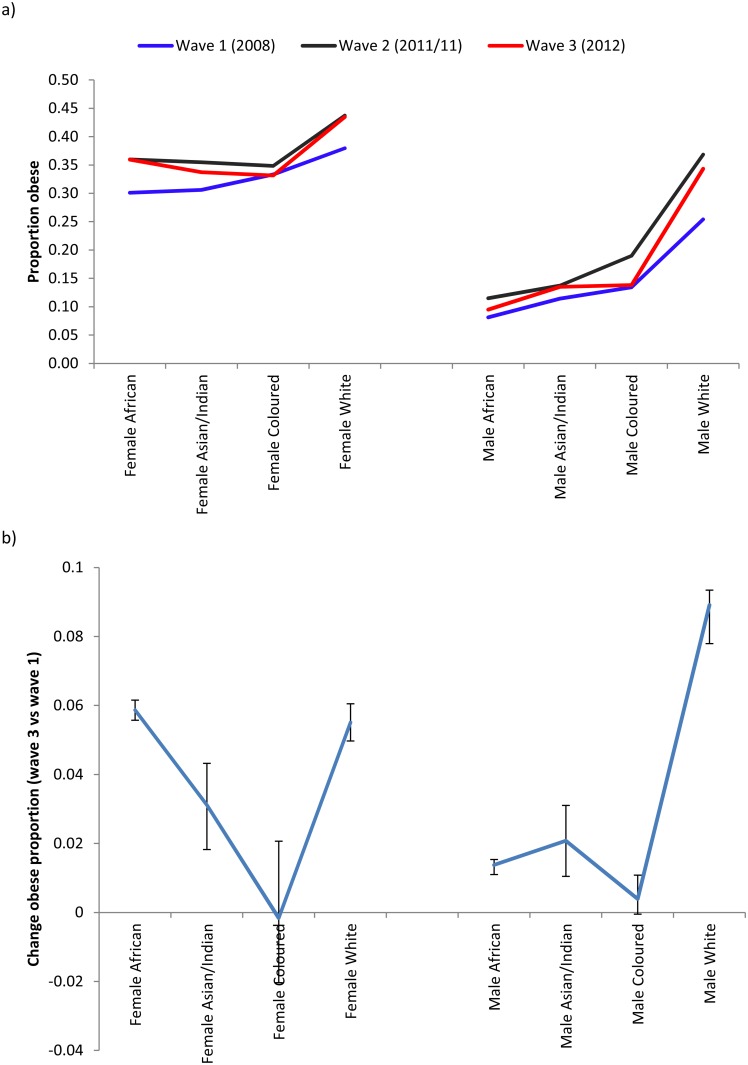
**a)** Trends in obesity prevalence by ethnicity, gender and period b) Change in obesity prevalence by ethnicity and gender comparing 2012 to 2008.

Further ethnic/gender specific analyses of obesity level and change over the 3 panels can be observed in Fig 3a and 3b. The highest level of obesity among females was observed amongst Caucasians in wave 1 followed closely by Africans. The relative gain in obesity from wave 1 to wave 3 was slightly greater however amongst Africans. The highest levels of obesity as well as the greatest gain from 2008 to 2012 were observed amongst Caucasians.

The following factors were associated with increased odds of obesity among males following multivariable adjustment (Table 3): formal urban residence (odds ratio [OR] = 1.71, 95%CI = 1.22–2.40) based on rural residence as the reference category, white ethnicity (OR = 2.26, 95% CI: 1.44–3.54), married, belonging to the high/highest socio-economic quintiles (OR = 1.68 and 1.32 respectively) and/or lack of exercise (OR = 1.24, 95%CI: 1.04–1.48). Conversely male smokers were significantly less likely to be obese.

**Table 3:** Variables associated with obesity among South-African adults (≥15 years) using a generalised linear latent and mixed model multilevel logistic regression, 2008–2012.

	Men				Women			
	Bivariate		Multivariable		Bivariate		Multivariable	
Characteristic	Odds Ratio (95% CI)	p-value	Odds Ratio (95% CI)	p-value	Odds Ratio (95% CI)	p-value	Odds Ratio (95% CI)	p-value
**Lifestyle/Behavioural (individual)**								
**No exercise**								
No	1		1		1		1	
Yes	1.32 (1.19, 1.47)	<0.001	**1.24 (1.04, 1.48)**	**0.015**	1.17 (1.08, 1.25)	<0.001	**1.09 (1.04, 1.14)**	**0.001**
**Current smoker**								
No	1		1		1		1	
Yes	0.56 (0.49, 0.64)	<0.001	**0.37 (0.3, 0.45)**	**<0.001**	0.65 (0.58, 0.74)	<0.001	**0.7 (0.64, 0.77)**	**<0.001**
**Drinks alcohol**								
No	1		1		1		1	
Yes	0.95 (0.85, 1.06)	0.387	---	---	0.76 (0.7, 0.83)	<0.001	**0.9 (0.85, 0.96)**	**0.001**
**Depressed**								
No	1		1		1		1	
Yes	0.90 (0.80, 1.00)	0.065	1.12 (0.93, 1.35)	0.232	1.12 (1.05, 1.20)	0.001	**1.04 (1, 1.08)**	**0.063**
**Household expenditure on food (household)**								
Low	1		1		1		1	
Medium	1.27 (1.12, 1.45)	<0.001	1.01 (0.82, 1.25)	0.925	1.23 (1.15, 1.31)	<0.001	**1.12 (1.08, 1.17)**	**<0.001**
High	2.13 (1.88, 2.42)	<0.001	1.21 (0.96, 1.53)	0.113	1.49 (1.38, 1.6)	<0.001	**1.18 (1.12, 1.24)**	**<0.001**
**High expenditure on unhealthy food options** ^i^ **(household)**								
No	1		1		1		1	
Yes	1.16 (1.05, 1.29)	0.005	0.95 (0.8, 1.14)	0.585	1.11 (1.04, 1.17)	0.001	**1.05 (1.01, 1.09)**	**0.009**
**Individual socio-demographic factors**								
**Age**								
15–24	1		1		1		1	
25–34	2.11 (1.75, 2.55)	<0.001	**2.14 (1.59, 2.88)**	**<0.001**	2.61 (2.36, 2.89)	<0.001	**2.01 (1.85, 2.18)**	**<0.001**
35–44	3.84 (3.17, 4.65)	<0.001	**3.64 (2.58, 5.15)**	**<0.001**	4.77 (4.28, 5.31)	<0.001	**2.82 (2.59, 3.07)**	**<0.001**
45–54	4.78 (3.94, 5.80)	<0.001	**5.47 (3.71, 8.07)**	**<0.001**	5.59 (5, 6.25)	<0.001	**3.06 (2.8, 3.35)**	**<0.001**
55–64	5.61 (4.55, 6.92)	<0.001	**6.38 (4.17, 9.77)**	**<0.001**	5.24 (4.64, 5.93)	<0.001	**3.08 (2.79, 3.39)**	**<0.001**
65+	4.27 (3.39, 5.38)	<0.001	**4.05 (2.52, 6.51)**	**<0.001**	3.49 (3.07, 3.97)	<0.001	**2.58 (2.31, 2.88)**	**<0.001**
**Population group**								
African	1		1		1		1	
Coloured	1.34 (1.12, 1.61)	0.001	0.91 (0.65, 1.28)	0.599	1.04 (0.94, 1.16)	0.416	0.99 (0.92, 1.06)	0.697
Asian/Indian	1.2 (0.7, 2.08)	0.509	0.61 (0.27, 1.4)	0.247	0.81 (0.56, 1.16)	0.246	**0.65 (0.51, 0.83)**	**0.001**
White	3.99 (3.23, 4.93)	<0.001	**2.26 (1.44, 3.54)**	**<0.001**	1.12 (0.93, 1.35)	0.238	**0.79 (0.7, 0.9)**	**<0.001**
**Education**								
No education	1		1		1		1	
Primary	0.95 (0.77, 1.19)	0.681	1.15 (0.8, 1.64)	0.450	1.17 (1.05, 1.3)	0.006	**1.16 (1.08, 1.24)**	**<0.001**
Secondary	1 (0.83, 1.22)	0.970	**1.62 (1.13, 2.33)**	**0.009**	0.82 (0.74, 0.9)	<0.001	**1.2 (1.12, 1.29)**	**<0.001**
Tertiary	3.12 (2.21, 4.39)	<0.001	1.75 (0.92, 3.35)	0.089	1.19 (0.93, 1.53)	0.163	1.06 (0.92, 1.24)	0.416
**Employed**								
No	1		1		1		1	
Yes	1.89 (1.69, 2.12)	<0.001	**1.46 (1.19, 1.8)**	**<0.001**	1.37 (1.27, 1.47)	<0.001	1.02 (0.98, 1.07)	0.347
**Social factors (family/household)**								
**Marital status**								
Married	1		1		1		1	
Living with partner	0.39 (0.31, 0.47)	<0.001	**0.46 (0.33, 0.64)**	**<0.001**	0.46 (0.4, 0.52)	<0.001	**0.78 (0.72, 0.85)**	**<0.001**
Widow/Widower	0.39 (0.26, 0.58)	<0.001	**0.32 (0.18, 0.58)**	**<0.001**	0.72 (0.65, 0.8)	<0.001	**0.87 (0.82, 0.93)**	**<0.001**
Divorced or separated	0.63 (0.43, 0.92)	0.018	0.6 (0.33, 1.1)	0.098	0.83 (0.69, 1)	0.056	**0.87 (0.79, 0.96)**	**0.006**
Never married	0.23 (0.2, 0.26)	<0.001	**0.34 (0.26, 0.45)**	**<0.001**	0.37 (0.34, 0.4)	<0.001	**0.8 (0.76, 0.84)**	**<0.001**
**Income quintile**								
Lowest	1		1		1		1	
Low	1.06 (0.87, 1.28)	0.556	1.06 (0.8, 1.41)	0.664	0.95 (0.87, 1.03)	0.181	**0.94 (0.89, 0.99)**	**0.019**
Middle	1.28 (1.06, 1.54)	0.009	1.09 (0.82, 1.46)	0.538	1.13 (1.03, 1.23)	0.006	1.03 (0.97, 1.09)	0.313
High	1.79 (1.5, 2.14)	<0.001	**1.51 (1.13, 2)**	**0.005**	1.27 (1.16, 1.39)	<0.001	**1.07 (1.01, 1.14)**	**0.018**
Highest	3.39 (2.86, 4.01)	<0.001	**2.04 (1.49, 2.78)**	**<0.001**	1.53 (1.39, 1.68)	<0.001	**1.11 (1.04, 1.19)**	**0.002**
**Environmental factors (community)**								
**Residence**								
Rural formal	1		1		1		1	
Tribal authority areas	0.85 (0.69, 1.04)	0.116	1.13 (0.79, 1.63)	0.498	1.18 (1.05, 1.34)	0.007	1.04 (0.96, 1.13)	0.37
Urban formal	1.61 (1.31, 1.96)	<0.001	**1.71 (1.22, 2.4)**	**0.002**	1.48 (1.3, 1.67)	<0.001	**1.16 (1.07, 1.26)**	**<0.001**
Urban informal	1.04 (0.77, 1.4)	0.793	1.54 (0.95, 2.5)	0.082	1.45 (1.22, 1.71)	<0.001	**1.22 (1.09, 1.36)**	**<0.001**
**Crime common** ^ii^								
No	1		1		1		1	
Yes	0.65 (0.46,0.93)	0.019	0.6 (0.31, 1.14)	0.116	1.57 (1.28, 1.92)	<0.001	**1.21 (1.06, 1.38)**	**0.005**

^i^: upper 75^th^ percentile of household money spent on sweets, cakes, sugar sweetened beverages, fast food scaled by total household expenditure on food;

^ii^: theft or burglary—overall proportion of households in a given sample cluster (sub-district) who indicated frequent

The following factors were associated with increased odds of obesity among females following multivariable adjustment (Table 3): formal or informal urban residence (OR = 1.37, 95%CI = 1.20–1.57 and OR = 1.49, 95%CI = 1.25–1.79), African ethnicity, being married, belonging to the middle- highest socio-economic quintiles, lack of exercise, high total household food expenditure and expenditure on unhealthy food options and/or crime. Conversely female’s smokers were also significantly less likely to be obese (OR = 0.87, 95%CI: 0.80–0.94).

**Fig 4:**
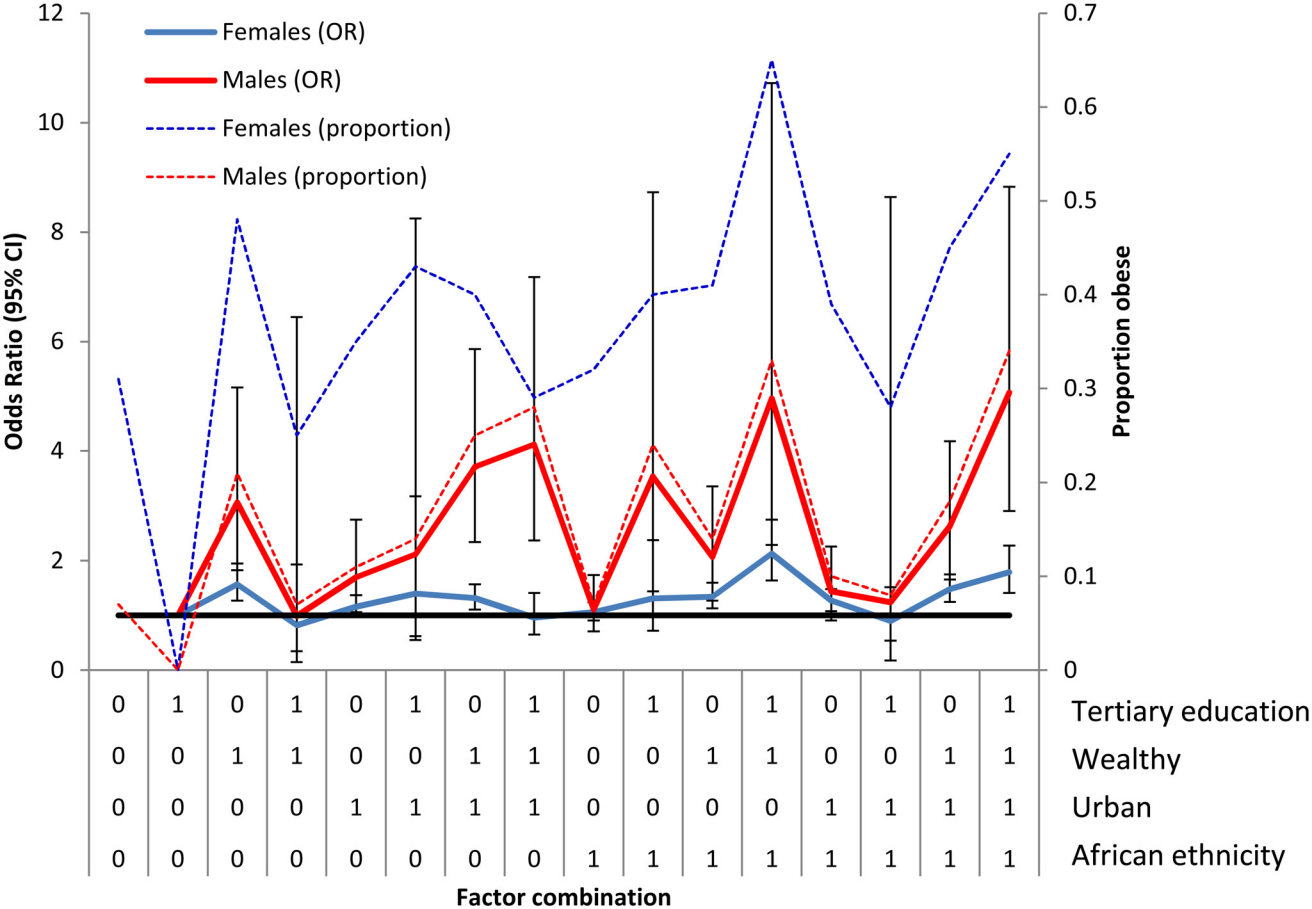
Interactions between significantly identified factors by gender (0 = No, 1 = Yes for each factor) and observed prevalence of obesity (secondary axis).

Interactions (sub-group analyses) by gender between selected significant covariates were also assessed and presented in Fig 4. The interaction analysis suggests that wealthy and tertiary educated African females/males were at the highest significant risk of obesity followed by the same demographic but additionally in an urban setting (compared to the reference group of non-wealthy, rural, non-tertiary educated, African females/males). The highest prevalence of obesity was also observed in this group. Wealthy, tertiary educated, urban non-African females were no longer at significant increased odds of obesity. Wealthy, white males (both tertiary and non-tertiary educated) were also at significantly elevated odds of obesity. Wealth among rural and urban African males remained a significant predictor of obesity (compared to the reference group of non-wealthy, rural, non-tertiary educated, African males) (Fig 4).

The population attributable fractions suggest after adjustment for confounding, lack of exercise among females and males potentially accounts for 7% and 9% of obesity respectively (Table 4). Amongst males, marriage appeared to be the most attributable determinant (24%) followed by urban residence (16%). At a population level marriage was a far higher attributable determinant among males compared to females (7%). Amongst females urban environment appeared to be the most attributable determinant (8%) followed by African ethnicity (7%). Proportionately high household food expenditure plus expenditure on unhealthy food options accounted for 6% of obesity amongst females. Seven percent of obesity among females was attributable to crime.

**Table 4:** Proportions of cases exposed to identified significant determinants and estimated population attributable fractions among South-African adults (≥15 years), 2008–2012.

Determinant	Total (obese)	Proportion of obese exposed [pd] (95%CI)^i^	Adjusted ^iii^ RR (95% CI)	AF*p*(95% CI)
**Males**				
No exercise	1876	0.60 (0.57, 0.64)	1.17 (1.06, 1.29)	0.09 (0.03, 0.14)
White ethnicity	1887	0.18 (0.11, 0.25)	1.32 (1.09, 1.59)	0.04 (0.01, 0.09)
Urban residence	1876	0.74 (0.69, 0.79)	1.29 (1.14, 1.45)	0.16 (0.09, 0.24)
Married	1882	0.59 (0.54, 0.64)	1.68 (1.48, 1.92)	0.24 (0.17, 0.31)
Wealth ^ii^	1800	0.46 (0.40, 0.53)	1.32 (1.17, 1.48)	0.11 (0.06, 0.17)
High household food expenditure	1887	0.50 (0.44, 0.56)	1.27 (1.12, 1.44)	0.11 (0.05, 0.17)
Total	---	---	---	0.75 (0.36, 0.95)
**Females**				
No exercise	9299	0.80 (0.78, 0.83)	1.1 (1.04, 1.15)	0.07 (0.03, 0.11)
African ethnicity	9328	0.82 (0.77, 0.87)	1.09 (1.02, 1.16)	0.07 (0.01, 0.12)
Urban residence	9300	0.62 (0.56, 0.68)	1.16 (1.10, 1.21)	0.08 (0.05, 0.12)
Married	9309	0.40 (0.37, 0.44)	1.22 (1.16, 1.27)	0.07 (0.05, 0.09)
Wealth ^ii^	8992	0.26 (0.22, 0.30)	1.06 (1.01, 1.11)	0.01 (0.00, 0.03)
Crime	9231	0.43 (0.40, 0.45)	1.21 (1.06, 1.37)	0.07 (0.02, 0.12)
High household food expenditure	9326	0.40 (0.37, 0.43)	1.14 (1.09, 1.19)	0.05 (0.03, 0.07)
High expenditure on unhealthy food options	9326	0.28 (0.26, 0.30)	1.05 (1.01, 1.08)	0.01 (0.00, 0.02)
Total	---	---	---	0.43 (0.21, 0.66)

^i^: Survey weighted;

^ii^: upper SES quintile;

^iii^: after multivariable adjustment

The summed AF*p*’s for selected determinants presented in Table 4 below suggest that they potentially account for ~75% of obesity among males and only ~43% in females.

## Discussion

### Prevalence of obesity

This study suggests that South African adults are undergoing a rapid epidemiological transition (as confirmed by the increasing obesity rates among both males and females) and South Africa has one of the highest prevalence’s of obesity in sub-Saharan Africa [4] at >27% (based on SA-NIDS survey estimates in 2012) and is in line with estimated from the South African Demographic and Health Survey in 2003 (based on backward trend trajectory of the SA-NIDS estimates)[32]. The high prevalence is largely due to females with 38% being classified as obese in 2012 based on this survey data. This is in agreement with recent findings which estimated that 31.8% of black South African females were obese [4]. By comparison only 13.3% of males were classified as obese. Many studies in South Africa [9,33,34] have also consistently reported a much higher prevalence of obesity among African women. This has also been observed in many other parts of sub-Saharan Africa, for example Botswana and Nigeria [35,36].

### Methodological framework

This study is the first to our knowledge to combine data from a national repeated panel study with rigorous statistical techniques, specifically the inclusion of population attributable fractions, to quantify and identify high impact determinants of obesity and thus best inform intervention strategies. Individual level data also allowed for discrete odds (“risk”) calculations for determinants (minimise confounding) as well as demonstrate potential interaction between determinants i.e. higher risk strata. This methodological framework can be easily adapted to other settings.

### Preventable attributable determinants of obesity and recommendations

#### Ethnicity/gender

Our study confirmed female gender (especially Black/African and White ethnicity) as a key determinant of higher obesity risk in South Africa. However, high proportions/odds of obesity were also observed in selected White and African male groups. These are discussed below.

#### African females

A previous study in South Africans [33] in an informal urban settlement suggested that being nutritionally deprived as children and higher socio-economic status were key factors that might explain the higher obesity rates amongst females. Another possible reason for the higher prevalence of obesity among South African black women is related to body image and a preference for a larger body size [37]. Thus far most interventions have focused on weight reduction among the overweight and obese. However, both conditions are notoriously resistant to treatment, particularly amongst Africans. Programs that focus on early intervention during adolescence have been identified as having the most potential impact on obesity in black women [38]. “However, such interventions must be sensitive to cultural belief systems and values” [38] which is especially important in the African context with regards to body image. A recent study in South Africa suggested that “implementing healthy diet and life style messages” into existing programs (such as the HIV/AIDS prevention programs) may be beneficial with little additional cost [39]. A recent randomised control trial concluded that obesity prevention (“maintain, don’t gain”) might represent a particularly effective intervention strategy for overweight women [40]. Maintaining overweight through “a medium-intensity primary care-based behavioural intervention” may be an appropriate public health strategy for women [40]. Another study focusing on rural Black women specially suggests that inexpensive mobile technologies and existing health centre resources provide a viable platform for behavioural change interventions related to weight maintenance to reduce obesity-associated chronic disease risk [41]. Furthermore this type of strategy is less intensive and potentially more sustainable in developing settings than more resource intensive strategies aimed at weight loss [41].

#### African males

Our findings suggest that both rural and urban, wealthy, tertiary educated African males have high rates of obesity. The possible reasons underpinning this are urbanisation, increased SES, change in dietary patterns and reduced physical activity. This is discussed in more detail in the next section.

#### Non-Europeans and BMI cut-offs

It should be noted that cut-off points for the classification of obesity (BMI >30 kg/m2) have been developed and extensively validated among Europeans. However, its appropriateness in non-Caucasian populations has been questioned [42]. Previous studies suggest that the use of obesity cut-offs derived among Europeans may underestimate the cardio-metabolic risk associated with weight gain in other ethnic groups. Thus lower BMI targets may have to be used for assessment of obesity in certain non-European populations.

#### White females

The highest prevalence of obesity amongst white females was observed in the wealthy, non-tertiary educated, both urban and rural demographic. Lack of education may be a primary driver for this observed pattern as their educated counterparts were no longer at elevated risk of obesity. Education as a risk factor is discussed in a subsequent section of the discussion below.

#### White males

However the findings also clearly suggest that high and ever increasing obesity levels amongst White males may also be cause for concern. This demographic has almost 2.5 times higher obesity levels (34% vs 14%) compared to the next highest group (Coloureds) in 2012. Non-tertiary educated, rural, Wealth white males as well as their urban counterparts and those who are tertiary educated were at significantly elevated risk of obesity and demonstrated high obesity prevalence’s. SES, dietary habits and lack of physical activity as possible underlying reasons for these observed patterns are discussed in more detail in the subsequent section.

#### Urbanisation-socio-economic status and dietary intake-physical inactivity

Many studies in the developed world have demonstrated that higher socio-economic status is associated with reduced risk of obesity [43,44]. However in SSA and South Africa [9,33,34] the reverse has been shown to be true i.e. increased SES is associated with increased risk of obesity. This finding has been confirmed in this study (for both African and non-Africans) which demonstrated a significantly elevated risk of obesity among the higher socio-economic groups after adjustment for confounding influences e.g. population group, urbanisation and education status. Non-African (largely white), wealthy, highly educated females did not appear to have a significantly increased odds of obesity after stratification; however their male counterparts did (Table 4). As the economy and individuals earnings improve, they tend to adopt Western lifestyles decreasing their physical activity levels [45,46]. Increased wealth potentially contributes to this dietary shift to poorer food choices e.g. bigger portion sizes, and a more frequent intake of fast foods [i.e. animal fat, sugar and salt] and reduced intake of fruits, vegetables and grains that has been attributed to rising obesity levels in developing settings [47,48]. Our study also demonstrated that urbanisation (proxied by residing in urban areas) is an important determinant of obesity in South Africa. The most attributable determinant associated with female obesity in our study appeared to be urbanisation. “The increasing ‘obesogenicity’ of the environments external to individuals is likely to be the major driving force for the increasing obesity epidemic” [49]. Urbanisation generally results in the adoption of a more westernised lifestyle (poorer dietary habits and reduced physical activity). Urbanisation in South Africa (and elsewhere) has been shown to be linked to an increase in dietary fat intake [50]. Dietary intake (and quality thereof) has been shown to be associated with increased risk and prevalence of obesity and also forms part of the nutritional transition from rural to urban areas as discussed above. Our study confirms this as households in SA-NIDS who spent a higher proportionate amount on food stuffs placed individuals at a significantly higher risk of obesity.

Increase in physical inactivity and the adoption of a more westernised sedentary lifestyle with urbanisation have been shown to be major contributing factors to the increased rate of obesity in urban areas in Africa [51,52]. Our study has also suggested this as lack of exercise emerged as a prominent and attributable risk factors for adult obesity. Our population attributable estimates suggest that lack of exercise alone could account for 15% of obesity in South African adults. As theoretically 100% of obesity can be attributed directly to inactivity and diet, we cannot discount the effect of regression dilution bias and quality of reported exercise by the respondents. The 2003 SADHS has suggested that half the South African population were not sufficiently active [32]. Estimates from this study suggest that 60% of obese males and 80% of obese females do not exercise, though this does not include other aspects of physical activity e.g. occupational or household labour etc. Furthermore previous studies in South Africa have also demonstrated a differential in terms of physical activity and urbanisation i.e. reduced physical activity levels in urban compared to rural areas [32].

Therefore future obesity prevention programs in developing transitioning settings will need to first focus at the family level, targeting higher socio-economic individuals, especially in urban settings. A broad multi-faceted range of strategies are recommended: development of community-wide programs in multiple settings (e.g. homes, schools, workplace etc); influencing food suppliers to make healthy food choices more accessible and easier (again this should be in multiple settings e.g. supermarket chains, schools) and increase subsidisation for healthier food options through government fiscal policy; reducing the advertising (or marketing) of energy dense foods (“fast foods”) and beverages (sugar sweetened) for children, adolescents and adults; increased taxation of unhealthy food and beverage options through government fiscal policy; changing urban environments and public transport systems to promote physical activity; increased education and communications about the importance of healthy eating and increased physical activity [reducing television or sedentary time for example] to reduce chronic disease associated with obesity; and improved health care services (infrastructure) to manage currently overweight or obese individuals [49].

#### Education

Previous studies in South Africa and SSA have shown an association between level of education and obesity [35,53]. Our study has further confirmed that, after adjustment for socio-economic status i.e. wealth (generally highly correlated with education), some primary or secondary schooling was associated with increased risk of obesity when compared to those with no schooling. This should be interpreted with caution however as residual confounding from wealth may have influenced this finding. However a recent study again demonstrated that wealth was risk factor for obesity in women with lower education status, while women with higher education were protected [54]. In our study, individuals (both males and females) with tertiary level education were not at a significantly higher risk of obesity when compared to those with no schooling after multivariable adjustment (i.e. influence of associated higher SES taken into account). This suggests that the higher educated are the early adopters of healthier preferences and that SA is probably in transition towards the pattern of western countries, where obesity is more prevalent in lower SES strata i.e. it has been demonstrated that the burden of obesity in each developing country tends to shift towards lower SES groups as the country's gross national product increases [55]. Findings from the South African Demographic and Health Survey (DHS) in 2003 found a similar pattern in that women with no education and/or with a tertiary education had a lower BMI on average compared to those women with some schooling [32]. Thus preventions or intervention programs need to target populations living in low-middle SES circumstances with low-intermediate education levels who are exposed to increasingly obesogenic environments and who are susceptible to the default food options on offer [49]. Further investment in education programs is central to addressing this key determinant of obesity. Furthermore the health benefits of female education have been well documented for maternal and child health.

#### Marriage

Previous literature suggests that marriage promotes better health and increases longevity [56]. However in our study, marriage was identified as a prominent determinant of obesity and appeared to be more attributable for male obesity. Previous studies have similarly demonstrated the positive association between entry into marriage and obesity after adjustment for other potential confounding influences [56–58]. Married couples spend more time eating together often order readymade or fast food (unhealthy food options) while spending more time watching TV (sedentary) and exercising less. Conversely unmarried individuals spend a more time keeping fit (exercising) and eating less in an attempt to make themselves more attractive [58]. Obesity is thus likely to negate some of the positive longevity aspects associated with marriage. Conversely exit out of marriage (divorce or widower) is associated with a significant reduced likelihood of obesity in our study and also in line with a previous studies which have documented similar patterns [56] i.e. “adults may enhance their prospects in the marriage market by losing weight”[59]. The social implications of divorce however present their own problems. Interventions or strategies aimed at the “home environment” are needed to promote “healthy” marriages where a healthy diet and physical activity are actively adopted. Furthermore the implications for the cascade effect this may have on their child’s obesity potential (particularly from an environmental and behavioural perspective) can also not be discounted. A seminal study also demonstrated that obesity in parents is likely to contribute to obesity amongst their children both from directly (genetic) and indirectly (environmental) [60]. Strategies targeting the married couple could include the promotion of premarital nutrition education programs, couple-based physical activities, emphasis on controlled food portions for the family and broader communication (television and radio) programs emphasizing healthy eating patterns [58].

### Limitations

One key criterion for causality is temporality i.e. cause precedes the effect. “The potential for reverse causation bias is likely to be greatest in cross-sectional studies that capture symptoms and treatment episodes over longer time frames, such as the lifetime or past 12 months.” [61] Given that the primary study design is a repeated cross sectional study, we cannot discount the possibility of reverse causation bias. The relatively high number of missing or invalid weight/height measurements may have introduced selection bias and may affected both the internal validity (“distortion of a statistical analysis”) as well as the representativeness (external validity) of the findings in the broader South African context. As this was a secondary data analysis, certain determinants (e.g. daily dietary intake) were not captured in the primary study. One major methodological criticism of using population attributable fractions (AF_p_’s) is the bias that is introduced when measures of effect coefficients (directly used in the AF_p_ calculations) are not controlled or adjusted for other possible confounding factors [62]. Thus we cannot discount that potential residual confounding or under-adjustment may have affected the estimates produced in this study. However we did use one of the most internally valid approximations if confounding is present [25,29,30] and thus believe that this limitation is negligible.

## Conclusions

The prevalence of obesity has risen over the past 5 years and corresponds to a rising prevalence of Type 2 diabetes in South Africa [50,63], especially among females. This increase is potentially linked to certain key proxy indicators of higher risk such as gender and ethnicity, and alterable causes such as urbanisation, increased wealth and physical inactivity and increased (poor) dietary intake. Given that obesity is associated with significant health risks and comorbidities this has significant implications for public health and the economy in South Africa in coming years. Addressing the determinants of obesity will involve a multifaceted and multi-environmental strategy and will require prevention at the individual as well as at a population level. Despite cultural perceptions of attractiveness of larger body size amongst African females, targeting adolescent black African girls to prevent “future” obesity should be a priority and integrated into existing infrastructure such as primary health care centres and schools. Furthermore recent local evidence suggests that this cultural perception may be changing to a preference for more ideal or lower female weight amongst South African males [64](“Younger, **thinner** women with a lighter, yellower skin colour and a more homogenous skin tone [were] considered more attractive”) and has been demonstrated in their African-America counterparts in developed settings [65]. Previous literature also suggests that lower BMI cut-offs for metabolic disease may apply in other ethnic groups which is thus even of more concern. With rising costs in the private and public sector to combat obesity related NCDs, this analysis can inform the crafting of culturally sensitive mass communications and wellness campaigns. Understanding and knowledge of the social determinants is critical in order to develop “best buys”. There is an urgent need to continue to develop and evaluate population-based interventions for obesity, especially those that are environmentally focused.
